# Optimizing Vaccine Trials for Enteric Diseases: The Enterics for Global Health (EFGH) *Shigella* Surveillance Study

**DOI:** 10.1093/ofid/ofad586

**Published:** 2024-03-25

**Authors:** Kirsten Vannice, Calman Alexander MacLennan, Jessica Long, Andrew Duncan Steele

**Affiliations:** Enterics, Diagnostics, Genomics & Epidemiology, The Bill & Melinda Gates Foundation, Seattle, Washington, USA; Enterics, Diagnostics, Genomics & Epidemiology, The Bill & Melinda Gates Foundation, Seattle, Washington, USA; Enterics, Diagnostics, Genomics & Epidemiology, The Bill & Melinda Gates Foundation, Seattle, Washington, USA; Enterics, Diagnostics, Genomics & Epidemiology, The Bill & Melinda Gates Foundation, Seattle, Washington, USA

**Keywords:** capacity building, children, low- and middle-income countries, *Shigella*, vaccine

## Abstract

In this introductory article, we describe the rationale for the Enterics for Global Health (EFGH) *Shigella* surveillance study, which is largely to optimize the design and implementation of pivotal *Shigella* vaccine trials in the target population of infants and young children living in low- and middle-income countries. Such optimization will ideally lead to a shorter time to vaccine availability in the target population. We also provide a brief description of the articles included in the supplement.

## Teaser

This article describes the background and rationale for the Enterics for Global Health (EFGH) study, a multi-country surveillance study aimed at optimizing the design and implementation of pivotal *Shigella* vaccine trials.

*Shigella* is estimated to be the leading cause of diarrheal deaths after rotavirus globally, with an estimated 93 831 (95% CI, 35 860–185 931) deaths and 8.4 million (3.3–16.5 million) disability-adjusted life-years (DALYs) in children under 5 years of age attributed to *Shigella* in the Global Burden of Disease 2019 study [1]. Since 2004, the Bill & Melinda Gates Foundation has been investing in product development for vaccines to protect against shigellosis among children in low- and middle-income countries (LMICs), where burden is the highest and outcomes are most severe. Recently, substantial progress has been made in product development of O-antigen glycoconjugate vaccines, with several candidates moving through the clinical pipeline [2]. To ensure that vaccines can rapidly move into Phase 3 efficacy trials, the Enterics for Global Health (EFGH) *Shigella* surveillance study was initiated to support clinical site readiness and to generate key data that would inform optimal efficacy study design and end points. In this paper, we describe the rationale for investment in *Shigella* vaccine development and the EFGH study, which serves as a model for multipartner consortia and site-centered research.

## SHIGELLA BURDEN

*Shigella* has consistently been identified in epidemiologic studies as the leading cause of diarrhea, including moderate to severe and hospitalized diarrhea, in children above 1 year of age across LMICs in Africa, Asia, and Latin America. Two landmark diarrhea etiology studies, the Etiology, Risk Factors, and Interactions of Enteric Infections and Malnutrition and the Consequences for Child Health (MAL-ED) [3, 4] and the Global Enteric Multicenter Study (GEMS) [5, 6], conducted in 2009–2012 and 2007–2011, respectively, both estimated *Shigella*-attributable fractions of moderate to severe diarrhea >30% in children over 1 year of age. The rotavirus Vaccine Impact on Diarrhea in Africa (VIDA) study, conducted during 2015–2018 to evaluate changing etiologies following rotavirus vaccine introduction in the 3 African countries that also participated in GEMS, similarly found sustained *Shigella* burden. Nearly 20% of all moderate to severe diarrhea episodes were attributed to *Shigella* across children from 0 to 59 months of age, with higher burden in children >1 year compared with <1 year of age [7]. The Global Pediatric Diarrhea Surveillance system (GPDS), which determines the etiology of hospitalized diarrhea in 28 low- and middle-income countries around the world, estimated that in 2017–2018 13%–18% of hospitalized diarrhea in children aged 12–59 months was attributed to *Shigella*, with the highest burden of *Shigella* in Latin America, Africa, and South Asia [8]. These results, spanning the globe over a decade, all highlight *Shigella* as a critical cause of diarrhea, indicating that a vaccine could have a substantial impact on child health.

Furthermore, the burden of *Shigella* extends beyond direct diarrhea-associated morbidity and mortality. *Shigella* is more likely than other diarrhea etiologies to cause persistent diarrhea, which is associated with stunting and wasting [9]. Stunting, defined as linear growth >2 SDs below the median, is associated with negative impacts on cognitive development, risk of subsequent disease, and long-term economic potential [10]. *Shigella* infection has been shown to be associated with linear growth faltering [11–14]. The longitudinal cohort study MAL-ED estimated that even asymptomatic infection with *Shigella* led to a significant decline in linear growth up to 8 years postinfection [13]. In the GEMS study, *Shigella* diarrhea was associated with linear growth faltering, but only among those not treated with antibiotics [15]. The potential impact of antibiotics was further demonstrated through the 7-country AntiBiotics for Children with severe Diarrhea (ABCD) trial, which showed that children with watery diarrhea attributed to *Shigella* had improved outcomes, including reduced diarrhea duration and better linear growth, when randomized to a 3-day course of azithromycin [16]. Even in the context of effective antibiotic treatment, 1 modeling study estimated that over the 20-year period from 2025 to 2044, a total of 1.4 million deaths globally will occur from *Shigella,* of which approximately one-fourth will be related to *Shigella-*induced stunting [17].

In the context of poor etiologic attribution for most diarrheal episodes in low-resource settings, the World Health Organization (WHO) only recommends antibiotics when diarrhea is accompanied by blood in stool (suspected *Shigella*), or dehydration in children >2 years of age (suspected cholera); however, shigellosis mostly presents as watery diarrhea without dysentery. In MAL-ED, only 14.7% of children with shigellosis presented with dysentery [18], and thus they would go untreated under current guidelines with adverse consequence. In addition, antimicrobial resistance to *Shigella* is an important and growing threat. In 2017, the WHO highlighted *Shigella* as a medium-priority pathogen for new antibiotic research and development. In recent years, countries around the globe have seen outbreaks of drug-resistant *Shigella*, including resistance to first-line (ciprofloxacin) and second-line (azithromycin and trimethoprim-sulfamethoxazole) antibiotics. In Pakistan, over half of *S. flexneri* clinical isolates are resistant to amoxicillin/clavulanic acid (88%) and trimethoprim-sulfamethoxazole (77%); over a third of isolates are resistant to third-generation cephalosporin [19]. In Bangladesh, surveillance of children under 5 years of age presenting with diarrhea to 2 hospitals during 2001–2020 showed resistance to ciprofloxacin increasing from essentially 0% of *S. flexneri* and *S. sonnei* clinical isolates in 2001–2005 to >70% resistant in 2016–2020. Resistance was worse for *S. sonnei,* with >90% of isolates resistant to ciprofloxacin [20]. Similar trends have been observed for azithromycin and ceftriaxone, though to different degrees. *Shigella* isolates tested in the VIDA study in Mali, The Gambia, and Kenya showed high resistance to trimethoprim-sulfamethoxazole (95%), substantial resistance to ampicillin (48%), and low/no resistance to ceftriaxone, azithromycin, and ciprofloxacin [7]. While antibiotic resistance patterns differ in Africa, the trends in South Asia are a warning of what the future could hold for *Shigella* globally. As antibiotic resistance increases, *Shigella* infections will become harder to treat with more adverse outcomes.

## VACCINE PIPELINE

A number of *Shigella* candidate vaccines, the majority of which are O-antigen-based, are currently in clinical development [2]. To provide sufficient global coverage against shigellosis, there is consensus that these need to be multivalent in their final format, ideally targeting 4 key serotypes of *Shigella* (*Shigella sonnei* and *Shigella flexneri* 2a, 3a, and 6). Assessment in Phase 2 studies in the target population of LMIC infants and young children is accepted as the key prerequisite step to moving into Phase 3 studies [21] in EFGH sites.

The most advanced quadrivalent O-antigen-based vaccine is a bioconjugate vaccine developed by LimmaTech Biologics AG (Zurich, Switzerland) [22]. This has recently completed a Phase 2 age-descending dose-finding study in Kenya (NCT04056117), with results awaited. A second quadrivalent O-antigen-based vaccine, this time based on outer membrane vesicle technology, is currently being assessed in a similar Phase 2 study in Kenya (NCT05073003) by the GSK Vaccines Institute for Global Health [23]. A monovalent synthetic O-antigen conjugate vaccine from the Institut Pasteur has also been tested in a Phase 2 study in Kenya (NCT04602975), with results pending [24]. Concurrently, a quadrivalent version of this vaccine is being developed. The development of all 3 of these vaccines has received support from the Bill & Melinda Gates Foundation.

Other *Shigella* vaccines in clinical development include a bivalent glycoconjugate vaccine from the Beijing Zhifei Lvzhu Biopharmaceutical Company (Beijing, China) [25], currently being assessed in a Phase 3 study in China. Invaplex, a complex of *Shigella* lipopolysaccharide and invasion plasmid antigen (Ipa) proteins B and C, has been tested up to Phase 1 in several formats [26]. Finally, 2 live-attenuated *Shigella* candidate vaccines are currently in early clinical development: ShigETEC from Eveliqure Biotechnologies GmbH (Vienna, Austria) [27] and WRSS vaccines from the Walter Reed Army Institute of Research [28].

## THE ENTERICS FOR GLOBAL HEALTH STUDY

It is important that *Shigella* vaccines are made available to the populations most affected by shigellosis without unnecessary delay. EFGH was initiated to ensure that potential *Shigella* vaccine Phase 3 clinical trial sites are well characterized for *Shigella* incidence, severity, serotypes, and other factors that would influence efficacy study design. The EFGH study will generate data on children aged 6–35 months presenting to a health facility with diarrhea, with a focus on *Shigella*. The goal is to generate data on *Shigella* incidence for at least 2 years before the start of an efficacy trial across geographic regions. This will ensure high confidence in the design and sample size needed for successful Phase 3 trials. Most locations in the world do not have recent or robust estimates of *Shigella* incidence, which could lead to underpowered trials, and thus a failed vaccine. A second goal is to support site readiness through protocol and lab harmonization and capacity-building activities including staff training, such that any EFGH site could quickly initiate a multicountry efficacy trial when required. The EFGH sites are thus meant to represent a platform available for additional research and clinical trials that can be implemented without delay, ultimately accelerating access to a licensed vaccine. The data generated will also be important in characterizing the value of a *Shigella* vaccine beyond disease incidence alone by further characterizing antimicrobial resistance, cost of illness, disease sequelae, and more.

Separate investments have been provided for the EFGH study to target and optimize 3 components: (1) site selection, (2) planning and protocol development, and (3) study implementation. In May 2020, a solicitation for applications from potential EFGH study sites was targeted to leading principal investigators who had previously conducted diarrheal disease research studies in Asia, Africa, and Latin America. Applications were scored independently by 4 reviewers with scoring related to existing data on *Shigella* burden, ability to enumerate the catchment area population, experience with disease surveillance, ability to characterize care-seeking behavior, ability to enroll and retain children, history of diarrhea research studies, vaccine trial experience, electronic data capture, microbiologic laboratory capacity, and geographic representativeness. Of the 19 applications received (from 15 countries), sites were selected in 7 countries: Bangladesh, The Gambia, Kenya, Malawi, Mali, Pakistan, and Peru. Health care facilities in each EFGH country site were selected based on high volume of children presenting with diarrhea (ideally with demonstrated high burden of *Shigella*-attributed diarrhea), willingness to participate in a multicountry surveillance study, a patient population representative of the sociodemographic characteristics of the underlying catchment areas, and absence of current/planned diarrhea interventional trials at the time of EFGH recruitment. As highlighted in the supplement's site description papers [29–35], the sites represent a wealth of experience in diarrheal disease research, with all participating in 1 or more landmark diarrhea studies, such as GEMS, MAL-ED, and the ABCD trial [36].

**Figure 1.**
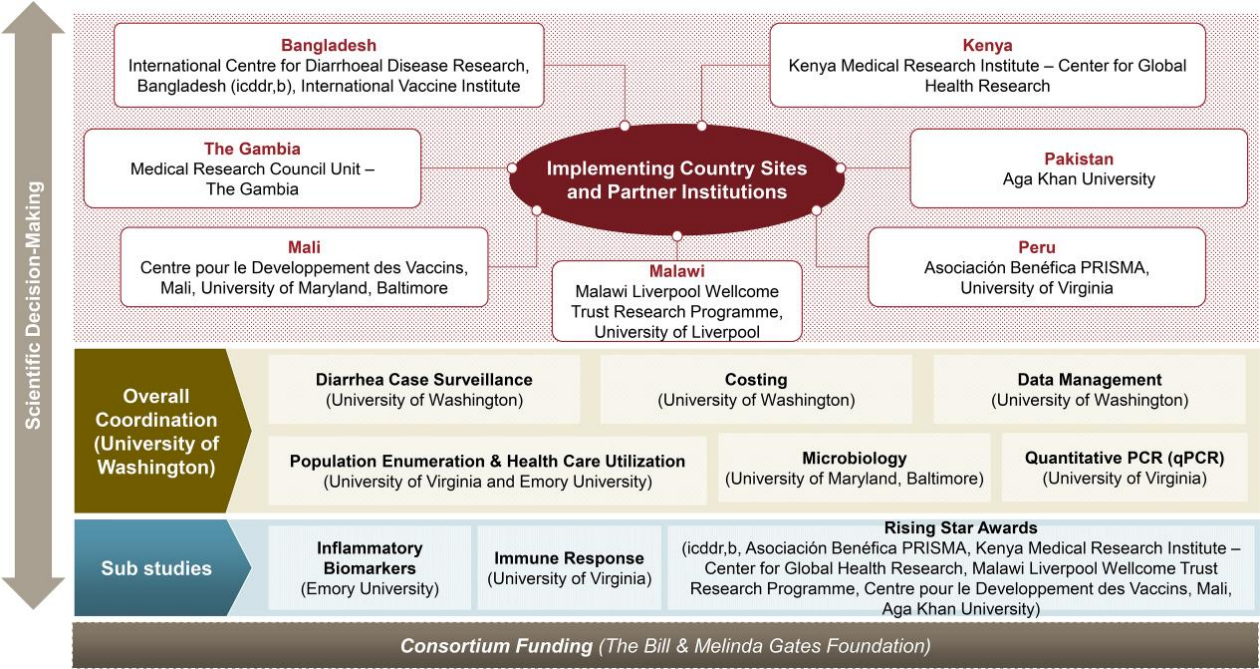
Governance structure of the Enterics for Global Health (EFGH) *Shigella* surveillance study.

A planning grant for protocol development was initiated after site selection to ensure scientific decision-making by site principal investigators (PIs) and site research teams, which all came with decades of relevant experience and expertise. Sites and coordinating teams were funded to plan and develop protocols over a 12-month period, enabling time to develop a strong scientific base to the study and ensure that time was not a barrier to collaborative engagement. A deliberate effort was made to define a governance structure that reflected the desire for all members of the consortium to have a voice and role in decision-making (Figure 1). As described throughout the supplement, the EFGH protocol and associated study procedures and documents were developed collaboratively across members of the consortium in biweekly domain-specific scientific working groups with interim review of documents. Wide engagement was facilitated by clear roles and responsibilities tied to the planning grant funding of site and coordinating investigators. The timing of the planning phase from late 2020 to 2021 also allowed for changes in care-seeking behavior and disease transmission affected by the SARS-CoV-2 pandemic to normalize. Close monitoring of manufacturer timelines was also maintained to align study timelines for data collection in EFGH with readiness for potential Phase 3 trial initiation.

The current investment for EFGH is the implementation phase, with the first enrollment of participants taking place on June 21, 2022. As described in this supplement, EFGH is a facility-based hybrid surveillance study enrolling children aged 6–35 months presenting with diarrhea at selected study health facilities [37]. Enrollment is over a 24-month period in order to document 2 full calendar years of shigellosis with *Shigella* detected by both traditional microbiologic methods [38] and molecular methods [39]. Enrolled children are followed longitudinally for 3 months to ascertain outcomes including diarrhea duration and recurrence, linear growth faltering, hospitalization, and death. The underlying catchment area populations are estimated with sampling and health care utilization continuously monitored over the 24-month period in order to estimate incidence rates of medically attended *Shigella* diarrhea [40]. Embedded in this rich study and data/sample repository are efforts to estimate the cost of *Shigella* diarrhea to families and health care systems [41], to establish immune response following *Shigella* infection [42], and to determine biomarkers of *Shigella* diarrhea that could be adapted to targeting strategies for antibiotics [43].

## EFGH BEYOND SHIGELLA

The EFGH represents a new opportunity to serve country sites in a deliberate and site-driven manner and to further principles of training and capacity-building both across and within the consortium. Site PIs and research teams are surveyed anonymously biannually to identify site-specific goals and opportunities as well as barriers to manuscript writing. Early career investigators (ECIs) have been consistently identified as a key priority for growth and support. ECIs named data analysis, protected time for writing, and pilot funding as key areas to support their career development. With the large infrastructure of EFGH to support secondary analyses and substudies and with a diverse network of experienced researchers, 2 site-focused ECI support programs have been developed, the EFGH Rising Stars Program and the EFGH Manuscript Writing Certificate Program. The EFGH Rising Star Seed Award [44] was modeled after the Global Center for Integrated Health of Women, Adolescents, and Children (Global WACh) Rising Stars awards [45] at the University of Washington; a first round provided between USD$21,000 and $30,000 for 3 early career scientists from 3 EFGH sites to conduct independent research mentored by senior site investigators. Additionally, these Rising Stars became nonresidential visiting scholars at the University of Washington's Global WACh center, giving them access to many of the institution's privileges, such as library access, database support, peer mentorship meetings, and networking and presentation opportunities. A second round of EFGH Rising Stars applications is underway, which should provide further research support and opportunities for ECIs. The complementary EFGH Manuscript Writing Certificate Program is a 16-month structured training and mentorship program intended to support first-author manuscripts led by ECIs from EFGH sites using already collected data from the EFGH study. Monthly meetings, work plans, and asynchronous lectures on study design, scientific writing, and data analysis, along with one-on-one mentorship from site and coordinating investigators and data analysts, comprise this program's unique approach to manuscript writing support. Equitable authorship is also a key goal of the EFGH, with the consortium papers in this supplement being co-written by site and coordinating teams, a process that included clear authorship roles and responsibilities, accountability mechanisms, and working group meetings where manuscripts were outlined and sections divided among the authorship group. Other activities, including University of Washington Global Health E-Learning courses [46] and external support for scientific editing, have also been made available.

## CONCLUSIONS

The EFGH Study is a large, multicountry consortium for which special emphasis has been placed on protocol development being undertaken jointly by the site leadership and coordination teams. This protocol supplement represents the culmination of over a year's work by consortium members. A description of each site is provided, which highlights the sites’ unique differences in population, health facility infrastructure, care-seeking, and site-specific emphasis on diversity, equity, and inclusion. Other papers detail key aspects of EFGH study design and execution, including diarrhea case surveillance, population enumeration and the health care utilization survey, microbiologic and molecular lab diagnostics, and cost of illness data collection [37–41]. Details seldom found in the published literature, such as on data management [47], are provided here to support cross-study learning. Two EFGH substudies, one focused on evaluation of serotype-specific serological assays for *Shigella* and the other focused on characterizing inflammatory biomarkers of bacterial infections that could in the future lead to better antibiotic treatment targeting, are also described [42–43]. We hope that the EFGH, with a focus on changing the global health research power dynamics through both funding pathways and decision-making, can be a model for the future, both in the diarrheal research space and beyond.
